# Heterogeneity of the COVID-19 Pandemic in the United States of America: A Geo-Epidemiological Perspective

**DOI:** 10.3389/fpubh.2022.818989

**Published:** 2022-01-26

**Authors:** Alexandre Vallée

**Affiliations:** Department of Clinical Research and Innovation, Foch Hospital, Suresnes, France

**Keywords:** COVID-19, COVID-19 pandemic, spatial heterogeneity, geo-epidemiology, COVID-19 transmission, public health

## Abstract

The spread of the COVID-19 pandemic has shown great heterogeneity between regions of countries, e. g., in the United States of America (USA). With the growing of the worldwide COVID-19 pandemic, there is a need to better highlight the variability in the trajectory of this disease in different worldwide geographic areas. Indeed, the epidemic trends across areas can display completely different evolution at a given time. Geo-epidemiological analyses using data, that are publicly available, could be a major topic to help governments and public administrations to implement health policies. Geo-epidemiological analyses could provide a basis for the implementation of relevant public health policies. With the COVID-19 pandemic, geo-epidemiological analyses can be readily utilized by policy interventions and USA public health authorities to highlight geographic areas of particular concern and enhance the allocation of resources.

## Introduction

The spread of the COVID-19 pandemic has shown great heterogeneity between regions of countries, e.g., in the United States of America (USA) (1–4). Geo-epidemiological differences between incidence, infection and mortality rates have been correlated with arrival time of the COVID-19 virus (5), population age structure (6), socio-economic development and population density (7), the health insurance system (8), climatic and meteorological determinants (9), and anti-contagion policies and health practices (10). Recent studies have shown that the geo-epidemiological distribution of the epidemic waves has been heterogeneous across countries (11, 12). Thus, geo-epidemiological analyses using data, that are publicly available, could be of major importance to help governments implement efficient health policies (13–15).

With the worldwide COVID-19 pandemic, there is a need to better highlight the geo-epidemiological variability in the trajectory of this pandemic in different worldwide areas. Indeed, the epidemic trends across areas can change completely at a given time. Dynamics include increasing trends, leveling off, stationary incidence patterns, and decreasing trends. Moreover, the growth could be marked by several modes depicting different pandemic waves (16). The subnational level of the epidemic curves within a country will display different trends over time. Because the type and intensity of public health policies can vary across space, classifying and summarizing the geo-epidemiological dynamics of the COVID-19 pandemic is essential for real-time public health policy making (17).

**Figure 1:**
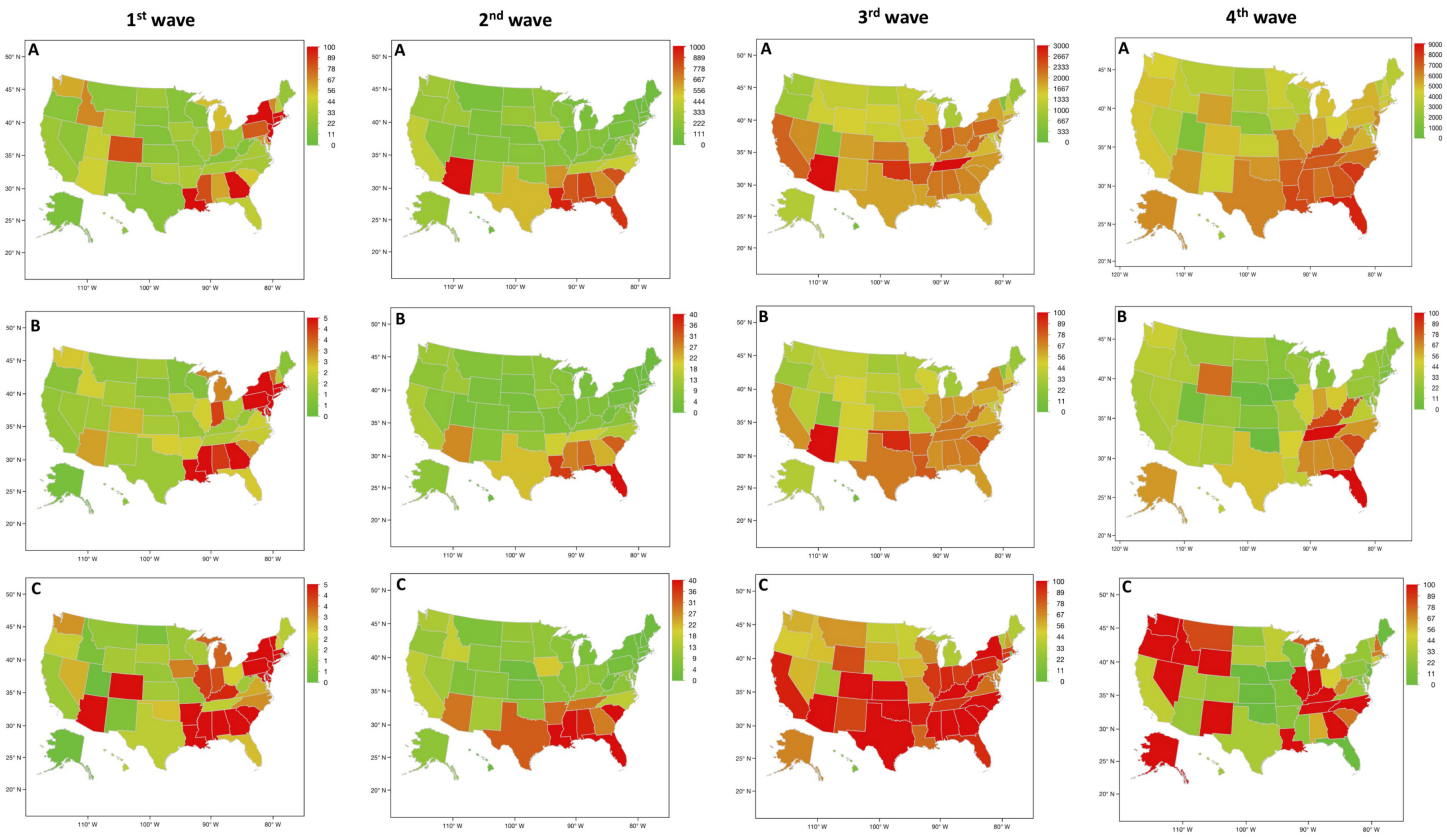
Count of new cases of COVID-19 patients across the USA for the four waves (new cases were reported by states divided by the population of each state and per 100.000 inhabitants). **(A)** New cases between the minimum of infection counts and the national peak time between two waves. **(B)** Rolling 7-day average before the national peak time of each wave. **(C)** Incidence of new cases for the national peak time of each wave.

The USA maps show no homogenous infection rates over time in regions regardless of the four waves (Figure 1A). On the day of the epidemic peaks for the waves, USA maps show very different infection rates between the regions on the day of the national peak regardless of the waves. This illustrates that local epidemic peaks appear at different times. The national peak may not be a good indicator for local management of the epidemic. Moreover, the distribution of new cases between the minimum of infection counts and the national peak time between the third and fourth wave shows that the fourth wave mainly developed in the southeast of the USA (Figure 1A, 4th wave) while during the national peak the states with the highest rates of infection were in the northwest (Figure 1C, 4th wave). Similar differences can be also observed for the rolling 7-day average before the national peak time of each wave (Figure 1B) and for the incidence of new cases at the day of the national epidemic peak (Figure 1C).

The data that support the observations of this study are openly available in https://github.com/CSSEGISandData/COVID-19.

## Discussion

Showing epidemic trends in advance may reveal more about the geographic risks and social and economic determinants which impact the mechanism of COVID-19 transmission, as well as how to respond to it. Several investigations have focused on the prediction of the epidemic trend of COVID-19 (18–21), but there are few reports of countries that report high-resolution, geo-epidemiological data. Aggregate (at the level of states- or country-wide) data of epidemics can be irrelevant when the local levels are not factored in the absence of geo-epidemiological data (22).

Geo-epidemiological analyses of epidemics could be described as the approach by which one compares epidemiological data of these epidemics across different geographical regions and populations, in the process identifying high geographical resolution (e.g., neighborhood or county/municipality level), environmental, geo-temporal, and socioeconomic factors. This approach provides valuable information about the global and regional burden of epidemics that could shape resource-planning, policy making, funding, healthcare considerations, and therapeutic intervention (23). Moreover, geo-epidemiology could prevent the errors and/or lack of practical (policy-related) consequences associated with low-resolution, spatial data, i.e., state- or country-level data.

Interdisciplinary analyses have been shown to investigate epidemics (24). The consideration of geo-epidemiology in the design of policies could improve the impact of such policies (24–26). Moreover, local geo-referenced and temporal data could unmask dynamic and complex associations influencing disease dissemination (27, 28). For example, investigations of the biogeography associated with rabies may detect markers promoting epidemic spread as well as barriers preventing dissemination (29). In geo-epidemiological investigations, disease mapping, disease clustering and individuals and ecological analyses are closely related (30).

A more comprehensive picture of a spatial problem can be achieved when the results of geographical aggregate-level data are combined with those at the individual level. Multilevel modeling, hierarchical and contextual analyses are phrases describing one of the various statistical methods in which this combination is allowed (31). Multilevel modeling is a powerful technique that can be utilized to determine how much of the ecological effect can be explained by variations in the distribution of individual-level risk factors. At this effect, attempts have been made to integrate this kind of analysis into geo- and social-epidemiology (32). There are also developments incorporating time changes along with spatial variation. Spatial models often lack transparency into the determinants of epidemiological dynamics. Thus, geo-epidemiological models are able to provide new insights into the spread of epidemics that are otherwise unavailable.

Geo-epidemiological differences in the trajectories of the COVID-19 pandemic in different worldwide areas should be investigated. Geo-epidemiological investigation can enhance an objective framework to investigate the epidemic geo-epidemiological dynamics in different areas within the same country. Functional data analyses have been utilized to investigate biologic mechanisms, stock market trends, weather patterns, underlying medical conditions, growth rates, and speech data. In the context of the COVID-19 pandemic, in China, clustering of curves was investigated to understand travel patterns of migrants (17, 33). Although attempts have been used to enhance the accuracy and validity of these estimates, the current data and algorithms focusing on domestic and international COVID-19 transmission are rather unclear, because of the spatiotemporal heterogeneity of the spread of the pandemic and poor understanding of its transmission processes. As an argument, only knowing the infection rate of a state as large as Texas, in the USA, or countries as extended as Russia and Chile do not facilitate cost-effective, site-specific and control measures. In contrast, it was analyzing actual geo-temporal-epidemiological data that it was discovered that many epidemics (including COVID-19) disseminate through preexisting connecting structures (e.g., road networks, airlines transports) (22, 27).

With the development of the epidemic, the spread of COVID-19 has gradually shifted from an imported case pattern to a local case pattern. Greater transmission risks observed in areas were in regions with low-detection capacity, high transportation, or economic connectivity to the epicenter of the outbreak.

Several studies have shown that the transmission of an infectious disease is modulated by viral characteristics and population susceptibility, as well as social and health conditions (34). Besides these common markers, the COVID-19 pandemic is also influenced by many specific markers, such as population age structure and human mobilities (19, 35).

Moreover, socioeconomic factors could show significant associations with COVID-19 transmission. The proportion of primary industries is found to be mainly correlated with the spatiotemporal mitigations of COVID-19 in provinces. The proportion of medical resources and rate of medical accessibility in an area have a major action in the prevention of infectious diseases (36, 37). Density of population, urban development, and access to transportation could also be correlated with COVID-19 transmission. Moreover, the risk of COVID-19 infection is also mainly modulated by the local population age structure (38).

In addition, the geo-epidemiological heterogeneity in the spread of infectious diseases comes from the socio-economic, and environmental differences among the geospatial units themselves (32). Compared with the possible climate associations implied by several investigations (39, 40), several investigations demonstrate that population density (41), and restrictions in health and mobility policies (42) have a considerable effect on the spread of COVID-19 pandemic. Mobility and connectivity (43), in association with high population density (44), can enhance the pandemic transmission more in terms of the geo-epidemiological differences, supported by research focused on USA county daily commute data (45) and mobility data for Boston (46).

In conclusion, geo-epidemiological heterogeneity of the COVID-19 pandemic has been observed in different USA regions. This observation could provide a basis for the implementation of geo-epidemiological analyses to influence public health policies. Thus, geo-epidemiology of COVID-19 transmission could be readily utilized by policy interventions and the USA public health authorities to highlight geographic areas of particular concern and enhance the allocation of resources.

## Data Availability Statement

The datasets presented in this study can be found in online repositories. The names of the repository/repositories and accession number(s) can be found below: https://github.com/CSSEGISandData/COVID-19.

## Author Contributions

AV had the original idea, performed the interpretation, wrote the article, and approved the final manuscript.

## Conflict of Interest

The author declares that the research was conducted in the absence of any commercial or financial relationships that could be construed as a potential conflict of interest.

## Publisher's Note

All claims expressed in this article are solely those of the authors and do not necessarily represent those of their affiliated organizations, or those of the publisher, the editors and the reviewers. Any product that may be evaluated in this article, or claim that may be made by its manufacturer, is not guaranteed or endorsed by the publisher.
